# The need for a rights-based public health approach to Australian asylum seeker health

**DOI:** 10.1186/s40985-016-0020-9

**Published:** 2016-08-22

**Authors:** Jo Durham, Claire E. Brolan, Chi-Wai Lui, Maxine Whittaker

**Affiliations:** 1 Faculty of Medicine & Biomedical Sciences, School of Public Health School of Public Health, The University of Queensland, Herston Road, Herston, Queensland 4006 Australia; 2 Dalla Lana School of Public Health, University of Toronto, Toronto, Canada; 3 College of Public Health, Medical and Veterinary Sciences, James Cook University, Townsville City, Australia

**Keywords:** Advocacy, Asylum seekers, Australia, Immigration detention, Health professionals, Human rights, Right to health

## Abstract

Public health professionals have a responsibility to protect and promote the right to health amongst populations, especially vulnerable and disenfranchised groups, such as people seeking asylum and whose health care is frequently compromised. As at 31 March 2016, there was a total of 3707 people (including 384 children) in immigration detention facilities or community detention in Australia, with 431 of them detained for more than 2 years. The Public Health Association of Australia and the Australian Medical Association assert that people seeking asylum in Australia have a right to health in the same way as Australian citizens, and they denounce detention of such people in government facilities for prolonged and indeterminate periods of time. The position of these two professional organisations is consistent with the compelling body of evidence demonstrating the negative impact detention has on health. Yet in recent years, both the Labour and Liberal parties—when at the helm of Australia’s Federal Government—have implemented a suite of regressive policies toward individuals seeking asylum. This has involved enforced legal restrictions on dissenting voices of those working with these populations, including health professionals. This paper outlines Australia’s contemporary offshore immigration detention policy and practices. It summarises evidence on asylum seeker health in detention centres and describes the government’s practice of purposeful silencing of health professionals. The authors examine how Australia’s treatment of asylum seekers violates their health rights. Based on these analyses, the authors call for concrete action to translate the overwhelming body of evidence on the deleterious impacts of immigration detention into ethical policy and pragmatic interventions. To this end, they provide four recommendations for action.

## Background

The power of the executive to cast a man into prison without formulating any charge known to the law, and particularly to deny him the judgment of his peers, is in the highest degree odious and is the foundation of all totalitarian government…. Nothing is more abhorrent than to imprison a person or keep him in prison because he is unpopular. That really is the test of civilisation. Winston Churchill, Nov. 21, 1943.

People seeking asylum often have complex physical and mental health needs. These may include infectious diseases not always seen in the host population, poor nutritional health and undiagnosed or untreated health conditions and injuries. In Australia, commonly observed physical conditions in asylum seekers include dental caries, digestive complaints, respiratory problems, skin lesions, dermatophytosis, otitis externa and infections of the upper respiratory tract [1]. Poor mental health is also common, as a result of this populace’s complex pre-arrival experiences, including torture and trauma and compounded by indefinite immigration detention on arrival [1]. As such, people seeking asylum require specific, comprehensive and consistent healthcare attention at the same level of quality as that provided to the citizens of the host country. Affirmative action is especially required to support the specific healthcare needs of newly arrived women and children.

In this paper, based on a comprehensive review of the literature, we outline Australia’s contemporary offshore immigration detention policy and practices and what is currently known about asylum seeker health in detention centres. While many countries detain illegal migrants, Australia’s policy of mandatory and indefinite detention, in which asylum seekers arriving by boat are detained in offshore processing centres, is one of the most restrictive immigration detention systems in the world. Furthermore, people are unable to legally challenge the need for their detention. The Australian detention policy has drawn criticism from health professionals as well as international bodies [2]. In this paper, we examine how this policy violates the rights of people seeking asylum, and the inter-related attempts of the government to purposeful silencing of health professionals. Based on the analysis, we call for concrete action: the translation of the overwhelming body of evidence on the deleterious impacts of immigration detention on the health, particularly the mental health, of people seeking asylum into ethical, common-sense policy and pragmatic interventions. As health professionals, we need to find innovative ways of raising the Australian public’s awareness of the government’s systematic derogation of its legal and moral responsibilities. To this end, we provide four recommendations at the end of the paper.

### Australian health policy for refugees and asylum seekers

In order to facilitate a better understanding of access to healthcare and the inter-related needs of refugees and asylum seekers, we outline here Australia’s current policy toward people seeking asylum (refer to the timeline). Refugees and asylum seekers can arrive in Australia either by aeroplane or boat. Many who arrive by aeroplane have usually been processed offshore by the Australian Government’s Department of Immigration and Border Protection when still in refugee camps in Africa, the Middle East, and Asia/South-East Asia. Such persons have subsequently been granted asylum prior to arrival in Australia by the Department of Immigration and Border Protection, which has deemed these persons to be genuine refugees as per the United Nations (UN) 1951 Refugee Convention and its 1967 Protocol (UN Refugee Convention). This particular group of asylum seekers are not subjected to mandatory immigration detention on arrival, and they are entitled to the same level of healthcare access as other permanent residents in Australia. This includes eligibility for Medicare and the Pharmaceutical Benefits Scheme, as well as access to interpreters.

### Timeline of major events in Australia’s mandatory detention policy

**Table:** 

Year	Key events
1992	• Introduction of mandatory detention limited to 273 days
1994	• Mandatory detention broadened to all non-citizens without a valid visa and the 273-day time limit removed • Introduction of Bridging Visas
1997	• Management of immigration detention centres outsourced to private companies
1998	• The Human Rights and Equal Opportunities Commission released the report *Those Who’ve Come Across the Seas: Detention of Unauthorised Arrivals*
1999	• Introduction of Temporary Protection Visas for refugees who arrive without authorisation
2001	• Introduction of the *Border Protection Bill* which provided the Australian Federal Government with the power to: - remove any ship in the territorial waters of Australia - use reasonable force to do so - provide that any person who was on the ship may be forcibly returned to the ship - guarantee that no asylum applications may be made by people on board the ship • *Migration Amendment Act* excises certain territories (among them Christmas Island) from the Australian migration zone • Implementation of the “Pacific Solution”: - introduction of offshore processing in Nauru and Papua New Guinea - excision of offshore territories from Australia’s migration zone - introduction of non-statutory refugee status determination process
2002	• Then UN High Commissioner for Refugees expresses concerns about the vilification of asylum seekers in Australia and urges the Australian Government to provide its citizens with accurate information • The UN Report on Mandatory Detention is released • The UN Working Group on Arbitrary Detention releases a scathing report on Australia’s detention centres
2004	• The Human Rights and Equal Opportunity Commission release its report *A Last Resort?* *National Inquiry into Children in Immigration Detention* • *AI-Kateb v Godwin*: High Court upheld the constitutional validity of indefinite detention • *Re Woolley*; *Ex parte Applicants M276/2003*: High Court upheld the constitutional validity of the mandatory detention of children • *Behrooz v Secretary, DIMIA*: High Court held that the harsh conditions of immigration detention did not render the detention unlawful
2005	• New “community detention” arrangements for families with children announced • Introduction of Removal Pending Bridging Visas for long-term detainees and those whose removal from Australia was pending but delayed • The UN Committee on the Rights of the Child recommends that conditions in Australian detention centres be brought up to international standards and that children be assessed within 48 h
2008	• Temporary Protection Visas abolished • The New Directions in Detention policy is announced • Offshore processing centres in Papua New Guinea and on Nauru are closed (end of the “Pacific Solution”)
2010	• Government announced plan to move significant numbers of children and their families out of immigration detention facilities and into community detention • *Plaintiff M61/2010E, Plaintiff M69/2010 v Commonwealth of Australia*: High Court held that any review of a refugee status assessment as part of an “offshore processing” regime is still bound by the provisions of the *Migration Act 1958* and decisions of Australian courts
2011	• Government announces arrangement to swap 800 asylum seekers from Australia for resettlement of 4000 refugees from Malaysia • *Plaintiff M70/2011 v Minister for Immigration and Citizenship*: High Court declares the arrangement with Malaysia invalid • Government announces the return to a single refugee status determination process
2012	• Expert Panel on asylum seekers delivers report making recommendations including the reinstatement of offshore processing • Offshore processing in Nauru and Papua New Guinea reinstated for those who arrived at an “excised offshore place” • “Enhanced screening process” introduced for all unauthorised maritime arrivals from Sri Lanka • The UN High Commissioner for Refugees released a report on the conditions in the offshore processing facility in Nauru
2013	• The UN High Commissioner for Refugees releases a report on the conditions in the offshore processing facility on Manus Island • Legislation passed to extend offshore processing to unauthorised maritime arrivals on the mainland • The Parliamentary Joint Committee on Human Rights released the report of its inquiry into changes introduced in response to the Expert Panel’s recommendations, concluded that “the measures as currently implemented carry a significant risk of being incompatible with a range of human rights” • A second UN High Commissioner for Refugees report focused on conditions in the offshore processing facility on Manus Island stated that “conditions remain below international standards for the reception and treatment of asylum seekers” • The UN Human Rights Committee found that Australia has breached the *International Covenant on Civil and Political Rights* by indefinitely detaining refugees who have failed security assessments
2014	• The *Migration and Maritime Powers Legislation Amendment Bill* is passed. Notable changes include: - power to detain people at sea (including outside Australia’s jurisdiction) and send them to other countries or vessels, even without the permission or knowledge of those countries - re-introduction of Temporary Protection Visas and the introduction of Safe Haven Enterprise Visas - introduction of a fast-track assessment process and removal of access to the Refugee Review Tribunal - creating a new statutory framework which sets out Australia’s own interpretation of its protection obligations under the UN Refugee Convention - retrospectively establishing the legal status of newborn children as “transitory persons” and “unauthorised maritime arrivals” - placing a “cap” on the number of Protection Visas that can be issued in any year
2015	• Australian Human Rights Commission releases its report, *The Forgotten Children: National Inquiry into Children in Immigration Detention* • The United Nations Special Rapporteur on the human rights of migrants, Francois Crépeau, called off his upcoming visit to Australia’s offshore detention centres
2016	• The High Court of Australia dismissed a challenge to the legality of the offshore processing regime. The government’s legal victory rested on a retrospective amendment to the *Migration Act* • Papua New Guinea’s Supreme Court rules that the transfer and detention of asylum seekers on Manus Island are both illegal and in breach of the right to personal liberty in the Papua New Guinean constitution. The Australian Government insists there is no chance these people will be resettled in Australia

Another group of individuals found to seek asylum in Australia include those who arrive by aeroplane with a legitimate visa to enter the country (i.e. business, tourist or student visa) and then later, often due to changing circumstances in their country of origin, apply for asylum “onshore”. This group of asylum seekers is also entitled to the same service or resource as the previous group (i.e. eligibility for Medicare and the Medicare and the Pharmaceutical Benefits Scheme, as well as access to interpreters). In contrast, asylum seekers who arrive by boat, mostly travelling from Indonesia, are deemed “Irregular Maritime Arrivals”. As the Irregular Maritime Arrivals have not arrived with a valid entry visa, they are considered by the Australian Government as illegal immigrants.

The Migration Act 1958 (Commonwealth) prescribes that any person who is in Australia without a valid visa must be detained, but there is no time limit on how long this detention may be. Since 1992, detainees have been held in onshore immigration detention centres, such as on Christmas Island or at Villawood in Sydney, or other closed detention facilities, known as immigration residential housing or transit accommodation. They may also be held in alternative places of detention (hospitals, psychiatric facilities, correctional centres, hotels) or in community detention. People in community detention (about 18 % of total immigration detainees as at 31 March 2016) can live unsupervised in the Australian community but are nonetheless legally deemed as remaining “in detention” [3]. Table 1

**Table 1:** Number of people in Australian immigration detention at 31 March 2016

Location of detention	All people^a^	%
Onshore detention in Australian mainland and territory		
Immigration detention centre/facility	1679 (17)	45.3
Community detention	655 (317)	17.7
Offshore detention in a third country		
Nauru detention centre	468 (50)	12.6
Manus Island detention centre	905 (0)	24.4
All forms of detention	3707 (384)	100

Source: Department of Immigration and Border Protection, *Immigration Detention and Community Statistics Summary*, 31 March 2016

^a^The number in brackets denotes the number of children in detention

provides a breakdown of number of people held in immigration detention. As at 31 March 2016, there was a total of 3707 people in immigration detention facilities or community detention in Australia and 384 of them were children [3].

**Fig. 1:**
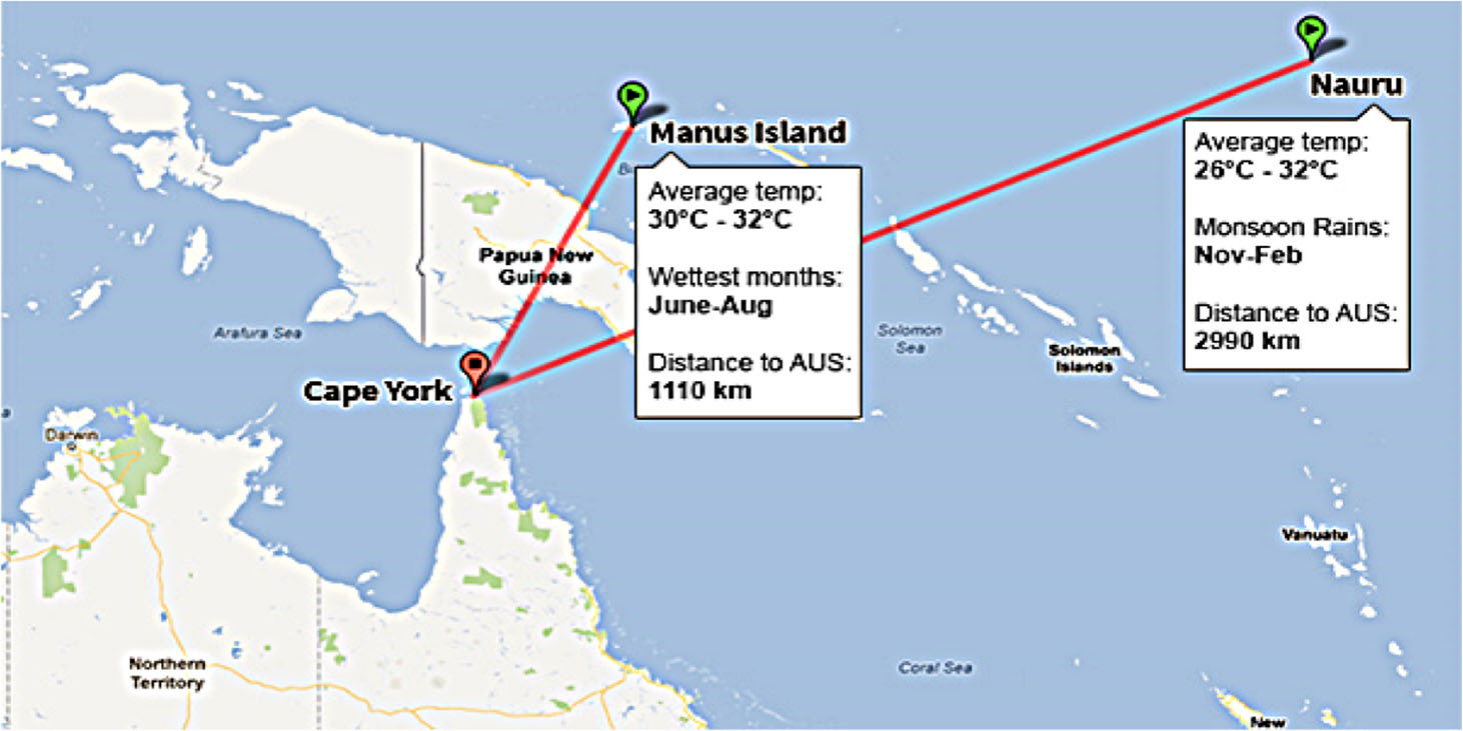
The Republic of Nauru and the Manus Island in Papua New Guinea. Source: http://www.news.com.au/national/offshore-processing-in-the-future-under-the-houston-review/story-fncynjr2-1226450008053

In 2001, the practice of transferring asylum seekers to offshore processing centres was introduced and subsequently re-introduced in 2012 when Federal Parliament passed amendments to the Migration Act 1958 (Commonwealth) that introduced a third country processing regime. These amendments stipulated that people seeking asylum, who arrive by boat, *must* be transferred to a third country as soon as it is reasonably practicable (with limited exception) [4]. Since then, asylum seekers arriving by boat are prevented from landing in Australia. Instead, these Irregular Maritime Arrivals may be turned around and returned to international waters or detained. If they are detained, they will be held in immigration detention facilities on Christmas Island, or transferred to offshore processing centres in the low-income Pacific island nation of Nauru (refer to Fig. 1).

Asylum seekers held in detention by the Australian Federal Government can be held indefinitely, with 454 days the average length of time a person spends in closed immigration detention [3]. As Table 2

**Table 2:** Length of time in held immigration detention facilities at 31 March 2016

Period detained	Total	% of total
7 days or less	82	4.9
8–31 days	185	11.0
32–91 days	176	10.5
92–182 days	189	11.2
183–365 days	319	19.0
366–547 days	185	11.0
548–730 days	112	6.7
Greater than 730 days	431	25.7
Total	1679	100

Source: Australian Border Force, *Immigration Detention and Community Statistics Summary*, various issues

shows, as at 31 March 2016, about 30 % (431) of the people held in detention facilities had been detained more than 2 years. Until recently, asylum seekers arriving by boat have also been taken to a naval base on Manus Island in Papua New Guinea. However, the Papua New Guinea’s Supreme Court ruled on 26 April 2016 that detention breached the right to personal liberty as embedded in the PNG constitution. Supported by the Papua New Guinea Prime Minister Peter O’Neill, the Papua New Guinea’s Supreme Court ordered that the Papua New Guinea and Australian Governments take immediate steps to end the detention of asylum seekers in Papua New Guinea [5]. While officially the approximately 850 men who were detained in Australia’s offshore processing centre on Manus Island are no longer in detention, they remain on Manus Island living in the detention centre with neither Australia nor Papua New Guinea taking responsibility for their resettlement. Information on how the healthcare needs of these people, of whom approximately 50 % have been recognised as refugees, are being met in this period of transition has not been reported.

Even if the asylum seekers on Manus Island are later found to be genuine refugees as per the terms of the UN Refugee Convention, they will not be granted the right to be settled in Australia. Instead, they may be settled in Nauru, Papua New Guinea, or Cambodia, which are countries already struggling to provide for the basic rights and welfare needs of their own populations, including the needs of children. The Australian Government’s position on the Manus Island detainees highlights its onus is on the politics of deterrence as opposed to politics grounded in international law.

The detention centres are managed by private companies contracted by the Australian Federal Government. These companies are also responsible for the management of healthcare facilities located in the centres. The contractors provide onsite primary level health care and liaise with local health care providers to provide emergency and acute care as well as clinical care that cannot be provided onsite. Nauru has weak, under-resourced healthcare facilities and there is a lack of transparency around how the health needs of detainees are met. This lack of transparency is further compounded by Federal Government contracts that prevent health professionals employed in the detention centres from advocating for the health needs and rights of the people under their care, and for whom they have a legal fiduciary duty (i.e. duty of care) toward [6]. Further in 2015, the government passed legislation making it illegal for employees at detention centres to disclose information about the camps to the media [6, 7].

### Impact of immigration detention on detainees’ health

The geographic isolation of Nauru (and the previous site of Manus Island off Papua New Guinea’s coast) coupled with the deliberate lack of transparency, make it very difficult to obtain accurate information on the health status of people in detention. Most of the published research that we located focused on asylum seekers who have not been processed offshore and are held in detention within Australia. For example, Correa-Velez and colleagues [8] in a retrospective audit of asylum seekers attending three specialist clinics in Melbourne found that the most common reasons for presentations were prescriptions (16 %) and health problems associated with musculoskeletal (27.1 %), respiratory (21.4 %), psychological (26.5 %), digestive (19 %), skin (12.2 %), endocrine, metabolic or nutritional (12.2 %) and cardiovascular (11.1 %) disorders. Another audit of 102 consecutive asylum seekers attending a clinic in Sydney in 2000–2001 found that psychological issues were the most common reason for presentation [9].

In 2005–2006, the most frequent physical conditions treated in asylum seekers at Australian immigration detention centres were dental caries, digestive complaints, respiratory problems, skin lesions, dermatophytosis, otitis externa and upper respiratory tract infections [1]. In addition, immigration detention has been found to be associated with health consequences including dental, mental health, musculoskeletal problems and lacerations [1]. Skin diseases such as eczema, fungal infections and impetigo as well as ear infections such as otitis media, which if untreated can result in hearing impairment, have also been observed in immigration detention facilities. These physical conditions are a likely consequence of the stress and poor living conditions within the detention centres [10, 11]. Also of note is that not only is there limited empirical data on asylum seeker health, but most of the work cited in this section is not contemporaneous.

Between 1 July 2010 and 20 June 2013, 12 deaths were recorded in immigration detention facilities, of which six were found to be suicide [12]. According to a Commonwealth Ombudsman report, major factors contributing to the rise of self-harm activities in the Australian detention network include the closed and overcrowded environment, an increase in both the length of detention and the number of people housed inappropriately together [13]. A 2015 review commissioned by the then Minister of Immigration and Border Protection found evidence of at least three rapes in the offshore detention centre on Nauru as well as numerous incidents of sexual assault, physical assault and sexual harassment including women being offered longer showers if they allowed security guards to watch them, women being propositioned for sex and offered marijuana or cigarettes in return [14]. A 2014 report found that between January 2013 and March 2014, there were 33 incidents of reported sexual assault (the majority involving children) in Australian network of detention centres [15].

A cogent body of evidence also demonstrates that immigration detention coupled with long periods of uncertainty associated with the processing of asylum claims increases levels of psychological stress and both causes and exacerbates mental health and other health issues and the likelihood of an individual self-harming [2, 16–22]. Available evidence shows that anxiety, depression, post-traumatic stress disorder as well as self-harm and suicidal ideation are common in this population [23]. A 2004 study examined the mental health of 10 detained families (14 adults and 20 children) who had been held for an extended period of time in different remote immigration detention facility in Australia. This study found that all the adults and children experienced at least one psychiatric disorder, with 26 disorders identified among 14 adults and 52 among 20 children [24]. Poor mental health has also been identified as one of the most common reasons for an immigration detainee presenting, or being transferred by the Department of Immigration and Border Protection, to an Australian hospital [25]. Drawing on a series of commissions of inquiry undertaken into detention centres, health professional observations and a small number of systematic studies that have been undertaken in detention centres, Silove and colleagues [2] concluded that prolonged and indefinite detention has direct and prolonged negative impacts on detainees’ mental and psychosocial health. Between January 2011 and February 2013, there were 4313 incidents of actual, threatened and attempted serious self-harm recorded in immigration detention facilities in Australia [26]. In May 2016, in the Nauru detention centre, one detainee self-immolated and died, while five days later another attempted self-immolation and four others self-harmed in the preceding 24 h [27]. As the Royal Australasian College of Physicians has pointed out, many of the health problems experienced by people seeking asylum are a result of their prima facie detention and cannot be adequately addressed by medical practitioners while they remain in this harmful environment [28].

Available evidence also shows that detention centres are especially harmful to young people and children, particularly infants and toddlers, and persons with intellectual or physical disability [16, 29]. Risks to children include deteriorating parental mental health and function, institutional policies that undermine parenting and family life, cumulative adverse environmental and safety risks, lack of protection from exposure to physical violence and mental distress in adults, including adult family members [30–32]. Alarmingly, children in detention are also found to be at significant risk of physical and sexual abuse and maltreatment [28]. Suicide attempts have been documented in young children and adolescents [24] with the rates of self-harming in adolescents up to 12 times higher than that of the general population [29]. A 2012 Commonwealth Review of Australia’s Immigration Detention Network arrived at a similar conclusion [33]. Similarly, in 2014, the Australian Human Rights Commission documented that children in prolonged detention were exposed to very high risks of physical and mental harm or injury, as well as suffered significantly higher rates of mental health disorders in comparison to their peers living in the general community [15]. The Australian Human Rights Commission Report on the health of children in Wickham Point, an onshore detention centre for asylum seekers, also found that children who had been held in Nauru were extremely traumatised as a result of the cumulative impact of a traumatic boat trip, movement to different onshore and offshore detention centres, as well as the fear of returning to Nauru [34].

Despite the documented poor mental health experienced by detainees, evidenced by the level of suicide and self-harm, and often as a result of the conditions of their detention, both the Australian Government and the general public seem indifferent to this. Indeed, in the most recent 2016 Federal election, both major parties continued to campaign on a platform of maintaining the current detention policy for asylum seekers. Continuing to detain people while knowing that it contributes to, and can be a direct cause of, mental illness in asylum seekers, further breaches asylum seekers’ rights to be treated with dignity; but again, the practice reiterates bipartisan support for immigration detention [2].

Poor mental health is further compromised by detainees’ lack of access to specialist mental health resources and support [25, 35]. While the Department of Immigration and Border Protection declared that asylum seekers are provided with a standard of care “broadly comparable with health services available within the Australian community” [35], the available evidence suggests otherwise. Amnesty International, for instance, found unsafe medical practices in detention centres, including rapid health assessments occurring in inappropriate conditions and basic follow-up health services that are non-compliant with Australian standards [36]. This includes inadequate antenatal care, including lack of access to ultrasound for pregnant women, inadequate medical care for children and poor prescribing practices and treatment of chronic conditions such as diabetes, delayed referral to tertiary levels of care, inadequate specialised mental psychiatric services, lack of access to adequate potable water and access to assistive devices for people with physical impairments [36].

## Discussion

### International law

Having examined the content of Australia’s contemporary offshore immigration detention policy and practices, as well as what is known about asylum seeker health in immigration detention facilities, we will now turn to investigate the violation of the right to health, and other human rights, of persons seeking asylum, and the inter-related silencing of Australian health professionals who provide healthcare to these persons. This is because the health and well-being of people seeking asylum are not a matter of Australian domestic public health policy only, but are complex matters coalescing with international law.

Australia and Nauru are parties to the UN Refugee Convention and a number of key international human rights law instruments. As parties to the Convention and other formative UN treaty documents, the holding of people seeking asylum indefinitely in immigration detention facilities, especially children, results in both the Australian and Nauru Governments being in breach of their obligations under international law. In so doing, this further escalates the vulnerability of persons within these immigration detention facilities to additional human rights violations [37].

For those adults and children placed in detention by the Department of Immigration and Border Protection in geographically isolated facilities on Nauru under Australia’s third country asylum policy, this makes a bad situation worse [14, 15, 38, 39]. This is not surprising, given these facilities are in a low-income country characterised by weak governance and whose government struggles to provide the basic necessitudes of life to its own populace [40]. The government of Nauru is also the recipient of substantial development aid from the Australian Government [40], and much of the population is reliant on Australia’s immigration detention centre for income. It is hard for the Nauru Government to refuse the requests of its more powerful regional neighbour. On the other hand, with Nauru a key partner in Australia’s asylum seeker detention policy, Australia has also been accused of failing to engage the Nauruan Government with issues of democracy and the rule of law. The subcontracting of health services in detention centres to for-profit companies has further exacerbated these issues [16]. In addition, issues of accountability, transparency and the conditions in which asylum seekers are detained have repeatedly been raised, including in an open letter by doctors working in Australian immigration detention facilities on Christmas Island [36].

Australia’s systematic breaches of its obligations and duties under international human rights law toward people seeking asylum, including their right to enjoy the highest attainable standard of physical and mental health (“the right to health”) [41], have been well-established [42, 43]. The recognition of the right to health as a fundamental human right imposes a tripartite typology of state obligations to facilitate equal access to health care services, to respect, and to protect people’s health regardless of their immigration status [44–46]. Indeed, a former UN Special Rapporteur on the Human Right to Health reiterated that the right to the highest attainable standard of health is to be enjoyed without discrimination (in line with General Comment 14 of the UN Committee on Economic, Social and Cultural Rights) and is especially important for vulnerable persons such as persons seeking asylum and in detention [47].

The right to health is based on human dignity and is codified in a number of interdependent international human rights treaties, predominately Article 12 of the International Covenant on Economic, Social and Cultural Rights, Article 25 of the Convention on the Rights of Persons with Disabilities, Article 12 of the Convention on the Elimination of All Forms of Discrimination against Women, Article 24 of the Convention on the Rights of the Child and Article 4 of the Convention on the Elimination of All Forms of Racial Discrimination. Importantly, Australia has ratified all of these international treaties and is obliged under paragraph 26 of the Vienna Convention on the Law of Treaties to act in good faith and implement the treaty provisions into its domestic law and policy [48]. According to this particular paragraph of the Vienna Convention, domestic law cannot be invoked as a legitimate reason for failure to abide by a treaty. Even if a person seeking asylum is not found to be a refugee under the terms of the 1951 UN Refugee Convention by the Australian Government or Australian court system, this individual’s dignity and right to health are still protected by the International Covenant on Economic, Social and Cultural Rights and related UN conventions, but also through Australia’s ratification of the Protocol against the Smuggling of Migrants by Land, Sea and Air, which protects the rights and humane treatment of smuggled migrants. This Smuggling Protocol is one of three protocols supplementing the Convention against Transnational Organised Crime, adopted by the UN General Assembly in 2000. It entered into force on 28 January 2004, and as of 10 July 2016, it has 112 signatories and 142 States Parties, with the Australian Government ratifying the Smuggling Protocol on 27 May 2004 [49]. The Protocol seeks to protect the rights of migrants particularly from the abuse and inhumane treatment of organised criminal groups, especially international or transnational criminal groups that work across country and maritime borders [50].

The UN Committee on Economic, Social and Cultural Rights has outlined in its General Comment 14 that the right to health espoused in Article 12(1) of International Covenant on Economic, Social and Cultural Rights contains two elements: the right to timely and appropriate health care and underlying determinants of health, specifically “access to safe and potable water and adequate sanitation, an adequate supply of safe food, nutrition and housing, healthy occupational and environmental conditions, and access to health-related education and information, including on sexual and reproductive health” [51]. As the evidence presented in this article forcefully demonstrates, both rights to health elements are being breached with respect to individuals in Australia’s immigration detention facilities overseas, and especially in regard to women, girls and all children. Notably, in 2014, the National Inquiry into Children in Immigration Detention [15], supported in 2015 by the Moss Review [14], found the conditions in which children were held in immigration detention facilities in Nauru and Papua New Guinea constituted a breach of their right to health, and other formative, inter-related rights, under the Convention on the Rights of the Child.

However, a number of national and international authorities have implicitly extended right to health breaches in the context of Australia’s offshore immigration detention system to encapsulate breaches of detainee’s right not to face inhuman and degrading treatment under Article 7 of the International Covenant on Civil and Political Rights, as well as the UN Convention against Torture and Other Cruel, Inhuman or Degrading Treatment or Punishment (Torture Convention) [36, 38, 39]. Tables 3 and 4 provide case examples. With respect to both of these examples, we note there were other non-health-related issues considered by the UN Human Rights Committee pertaining to both cases but, in the interests of parsimony, focused each vignette’s content on the Committee’s findings in relation to claimant’s allegations of human rights abuses on medical grounds.

**Table 3:** F.K.A.G et al. v Australia [111, 112]

*Decision by UN Human Rights Committee, established by the ICCPR; 20 August 2013* Facts: Thirty-seven Sri Lankan citizens arrived by boat and sought asylum were held in Australian immigration detention facilities, including excised to offshore detention locations. The claimants alleged the prolonged detention resulted in risks to their physical and mental health, and evidence of suicide attempts and inadequate medical treatment was tendered. The Australian government submitted that detention centres offered medical care comparable to that available to the general public, including mental health support services, and therefore there was not a violation of the claimant’s right to health, argued on the basis of Article 7 (right not to face torture, inhuman or degrading treatment or punishment) and Article 10(1) (failure to treat the detainees humanely and with respect for human dignity) of the ICCPR. Findings: The UN Human Rights Committee did not address the conditions of detention under Article 10(1) ICCPR. Instead, the Committee found although the Australian government provided access to medical treatment in detention facilities, Article 7 ICCPR with respect to the claimants had been violated. The Committee found medical services were “insufficient to rectify the negative impacts of prolonged detention on the mental states” of the claimants, and the “combination of the arbitrary detention, lack of awareness of future proceedings, and lack of information and procedural rights provided to the authors was cumulatively inflicting serious psychological harm. This harm constituted treatment contrary to Article 7 of the ICCPR”.

**Table 4:** M.M.M v Australia [113, 114]

*Decision by UN Human Rights Committee, established by the ICCPR; 20 August 2013* Facts: Nine claimants (two from Myanmar, six Sri Lankan, and one Kuwaiti citizen) arrived by boat between 2009 and 2010, and sought asylum. They were held in Australian immigration detention facilities. The duration of their detention seemed indefinite: although all claimants had been recognised by the Australian authorities as refugees as per the UN Convention on Refugees, they were denied visas for residence in Australia based on negative security assessments by the Australian Security Intelligence Organization (ASIO). The justifications and evidence related to the negative assessments were not conveyed to the authors. The claimants alleged the detention caused irreversible psychological harm and that the detention centre provided inadequate physical and mental health services. These allegations were confirmed in medical reports in relation to some of the claimants, and breaches of Article 7 (right not to face torture, inhuman or degrading treatment or punishment) and Article 10(1) (failure to treat the detainees humanely and with respect for human dignity) of the ICCPR were argued. Findings: The UN Human Rights Committee did not address the conditions of detention under Article 10(1) ICCPR. Instead, the UN Human Rights Committee found that Article 7 ICCPR in respect to the claimants had been violated. While the Australian government had presented evidence that the detention facilities provided mental and physical health services, the Committee found such services could not offset the negative impacts of ongoing detention for an indefinite duration.	

Indeed, as recently as March 2015, the UN Special Rapporteur on Torture, Juan Mendez, confirmed immigration detention of children as a form of “ill treatment” and that Australia’s asylum seeker policy of indefinite mandatory detention violated the Torture Convention [52]. Australia’s breach of the Torture Convention and Article 7 of the International Covenant on Civil and Political Rights not only derogates from the nation’s obligations under these two binding international law documents but also highlights Australia’s breach of its parallel duties under customary international law and peremptory jus cogens norms that wholly and utterly prohibit torture [53, 54].

### International law and medical ethics

As Australia’s offshore immigration detention facilities have been found to be torturous institutions by a number of prestigious and experienced international authorities, it would be unethical and potentially unlawful for Australia’s public health professionals to turn a blind eye [55, 56]. General silence and inaction is unconscionable—in fact, in breach of the foundational Hippocratic Oath and “Do No Harm” principle [57–59]. The interplay between international law, medical ethics, the conditions of detention and the contractual conditions of employees of detention providers also creates issues of conflict and tension for healthcare professionals [60]. While detainees have pressing healthcare needs, the conditions of detention are such that healthcare professionals are potentially colluding in deprivation of liberty [60, 61]. For those working in detention centres, the stipulation of their contract prevents them from being advocates for their patients in the interests of their employers. Refusing to work in offshore detention centres did not improve the conditions or the health of detainees either [60]. Further, healthcare professionals working in the detention centres do not have the capacity to address the underlying causes or the course of detainees’ ill health, particularly when related to mental health.

There is a myriad of sources that compel public health professionals to speak out in addition to professional and personal ethical frames of reference. These include the concepts of “beneficence” and “social justice” that partially underscore them, and include, but are not limited to, the Declaration of the Alma-Ata, Ottawa Charter on Health Promotion, the Committee on Economic, Social and Cultural Right’s General Comment 14 (see paragraph 62), and the UN Istanbul Protocol 1984 [62]. Health professionals advocating against immigration detention, therefore, do not do so merely as a matter of “moral outrage” as recently posited by an Australian media commentator [62, 63]. Rather, advocacy for the health of people seeking asylum, in the current Australian landscape, is a public health and human rights imperative and given the strong evidence base, an obligation of health professionals. Indeed, “public health is inherently political” [64] because “like any other resource or commodity… some social groups have more of it than others… its social determinants are amenable to political interventions and thereby dependent on political action (or more usually, inaction)….” [65]. Clearly, refugee health is markedly political [64]. Moreover, “Good health advocacy is not founded on hearsay—it is based on scientific evidence and the meritorious, well-documented concerns of health systems users” [64]. Consequently, Australia’s public health professionals’ advocacy is not simply moral outrage but is based on their reasoned concerns, given the evidence available and their professional obligations.

Given the foundational values of public health ethics, it is not surprising that Australia’s public health community has played an active role in challenging the Australian Government’s asylum policies and practices. The Public Health Association of Australia, Australian Medical Association, Australian Faculty of Public Health Medicine, the Royal Australian College of General Practitioners, and other peak health bodies have regularly and consistently asserted that people seeking asylum in Australia have a right to health in the same way as Australian citizens, and they denounced detention of such people in government facilities for prolonged and indeterminate periods of time [19, 66].

In 2015, fifteen peak health organisations in Australia, including the Australian Medical Association, the Australian Psychological Society, and the Royal Australasian College of Physicians, issued a joint statement requesting the Australian Government to immediately release all children and their families from immigration detention and to prevent further harm to children in their care [67]. Other examples of support by Australian health professionals in September–October 2015 have included several hundred staff at the Royal Children’s Hospital in Melbourne demonstrating against children in Australian immigration detention [63], reporting on the “refugee quandary” experienced by healthcare providers in the Medical Journal of Australia [68], and Professor John Ziegler’s petition in support of the Australian Human Rights Commission’s advocacy for children in immigration detention [69]. Healthcare practitioners in other paediatric hospitals across Australia, including Sydney, Darwin, Brisbane and Perth have also held protests against children in Australian immigration detention; in February 2016, the Lady Cilento Children’s Hospital in Brisbane refused to discharge an asylum seeker child back into detention [70]. Four-hundred and forty-five staff members of the University of Sydney also sent a petition to the new Australian Prime Minister Malcolm Turnbull in late September 2015, requesting he intervenes to stop the torture occurring in Australia’s offshore detention programmes [71]. Prominent domestic violence advocate and 2015 Australian of the Year, Rosie Batty, further called on Prime Minister Malcolm Turnbull to close Australia’s offshore detention centres [72]. More recently, a High Court challenge has been launched against the legislation that prevents employees at detention centres disclosing information about the detention centres on the basis that it breaches doctors’ constitutional freedom to engage in political communication and their duty to protect patients under their care [73].

### Where is the immigration health advisory group?

Health professionals’ and others’ recent vocalisation against Australia’s immigration detention policies mirror the concerns previously raised by the Immigration Health Advisory Group, formerly known as the Detention Health Advisory Group which was disbanded by the Australian Federal Government in December 2013 [74, 75]. The Detention Health Advisory Group was established in March 2006 as an independent advisory board to the Australian Government’s Department of Immigration and Citizenship (now Department of Immigration and Border Protection) [76]. It consisted of medical and public health professionals, psychiatrists, psychologists, nurses, dentists, general practitioners and others, including organisational representation of the Royal Australian and New Zealand College of Psychiatry, Royal Australian College of General Practitioners, Australian Medical Association, Royal College of Nursing Australia and the Public Health Association, with the Commonwealth Ombudsman having observer status [76, 77]. The Detention Health Advisory Group was created as part of a raft of “Palmer Plus” reforms undertaken by the Department of Immigration and Citizenship in response to two separate Commonwealth Inquiries into the unlawful detention in Federal immigration facilities of Cornelia Rau and Vivian Rau, both were Australian citizens [76, 78–80]. Following both Inquiries’ recommendations and advice from the Detention Health Advisory Group, the Department of Immigration and Citizenship launched a range of health-related activities that sought to improve the health and well-being of individuals in immigration detention. These included providing professional advice on the design, implementation and monitoring of health policy and procedures used in immigration detention [81], development of “Standards for health services in Australian immigration detention centres” by the Royal Australian College of General Practitioners [82], and involvement in scientific research led by the University of Wollongong into long-term effects of detention on the mental and physical health of immigration detainees [1]. According to the Department of Immigration and Citizenship in its Annual Report of 2011–2012, “DeHAG’s [the Detention Health Advisory Group] advice led to increased mental health staffing in facilities, as well as an external review of the implementation of the department’s psychological support programme, the key policy for managing self-harm risk in detention” [83]. Other activities arising from this relationship between the Detention Health Advisory Group and the Department of Immigration and Citizenship are reported in Table 5.

**Table 5:** Activities developed during 2006–2008/9 Detention Health Advisory Group advisory role to the Department of Immigration and Citizenship [76, 77, 81, 83, 84, 115, 116]

• Detention Health Advisory Group members visit and inspect places of immigration detention, including most active mainland places of immigration detention and Christmas Island. • Detention Health Advisory Group provides advice on the delivery of health services and the accommodation arrangements in place for children and people who have mental health or behavioural issues. • Detention Health Advisory Group Mental Health Sub-Group, created in March 2007, developed three new policies: o Identification and support of people in immigration detention who are survivors of torture and trauma. o Psychological support programme for the prevention of self-harm in immigration detention. o Mental health screening for people in immigration detention. • In introducing the above three policies to detention staff and other key stakeholders between February and August 2000, training was provided to approximately 1200 personnel from seven different government and non-government agencies and organisations that had extensive contact with people in immigration detention. • Detention Health Advisory Group Mental Health Sub-Group also provides advice to the Department on mental health-related professional development for staff working in the detention environment and developing a model for the management of mental health concerns for places of immigration detention in remote locations. • Detention Health Advisory Group Community and Public Health Sub-Group, created in 2010, provide independent expert advice in relation to public health issues and issues relevant to the health of clients living in the community—both as Bridging Visa holders and in community detention. • Detention Health Advisory Group Community and Public Health Sub-Group also provide guidance with respect to persons at risk of tuberculosis and to health care for minors in immigration detention.	

For several years, the relationship between the two was productive and collaborative, with the Department of Immigration and Citizenship describing the Detention Health Advisory Group’s experience and expert knowledge in shaping its responses to the challenges it faced “in a complex and sensitive programme area” [81]. This was recognised by the Department as an important step in promoting accountability and working with key health stakeholders in order to improve the general and mental health of detainees under the department’s care [84]. The overarching Immigration Detention Advisory Group, which was also represented on the Detention Health Advisory Group, further recognised the Detention Health Advisory Group’s important role and contributions [85]. Nevertheless, not all of the Detention Health Advisory Group’s concerns were addressed by the Department, as made clear by the Commonwealth Government of Australia’s Inquiry into Immigration Detention in Australia Joint Standing Committee on Migration’s Third Report of August 2009 [86]. This report also noted that the Detention Health Advisory Group had advised the Joint Standing Committee that it was only “an advisory body” with “no role in monitoring and no statutory right of entry to detention facilities” and, moreover, that it “was not set up [by the Department] to discharge the responsibilities of the immigration detention health review commission recommended by the Palmer Report” [86]. Therefore, the Joint Standing Committee noted the Detention Health Advisory Group was of the view the Palmer recommendation for an independent immigration detention health review commission should be implemented [86].

From its inception, the Detention Health Advisory Group played an important advocacy role for the health rights of people seeking asylum and other individuals in immigration detention. The Detention Health Advisory Group, together with other key interest groups such as the Australian College of Mental Health Nurses and the Australian Psychological Society, voiced its opposition toward mandatory detention and urged implementation of alternative existing solutions [87], especially with respect to the detention of children [88]. The Detention Health Advisory Group and its partner’s advocacy efforts were based on the clear scientific evidence of the harm caused by indefinite detention [89]. Yet, and around the same time, in its 2011–2012 Annual Report, the Department of Immigration and Citizenship flagged the Detention Health Advisory Group’s potential disbandment:

Since the establishment of DeHAG [the Detention Health Advisory Group], the number and composition of people in immigration detention, as well as the number and types of places of immigration detention, have changed considerably. IMAs [irregular maritime arrivals] now comprise more than 90 per cent of the detention population and significant numbers of clients are in the community under community detention arrangements, or as holders of bridging visas. Given the changing immigration detention environment, as well as changes in the way that health services are accessed by IMAs and other clients awaiting status resolution, a health advisory body focusing on health services in immigration detention facilities may no longer be appropriate. The department is therefore considering whether DeHAG should be replaced by a new health advisory body with a broader immigration health focus [83].

Subsequent to the above, the Detention Health Advisory Group was disbanded in August 2012 [90] and replaced by the Immigration Health Advisory Group in March 2013, and its focus was allegedly “broader” than the Detention Health Advisory Group [91]. However, by December 2013, the Immigration Health Advisory Group had also been disbanded by the Australian (Liberal) Government and replaced by a single Independent Health Advisor—whose independence was questioned in Australian media reports in light of the fact this sole position had now become a government role or that of departmental medical officer [92, 93].

In turn, the now-titled Department of Immigration and Border Protection’s official rationale for the Immigration Health Advisory Group’s disestablishment was that “a large representative body such as Immigration Health Advisory Group is less well equipped to do this [respond very quickly to our ever changing [government policy] environment] than an alternative panel capability that is able to respond to particular issues under consideration often within tight timelines, including on issues that might fall outside the current professional base of the IHAG [Immigration Health Advisory Group]” [90]. However, then Prime Minister Tony Abbott was less nuanced, reportedly stating “There was a committee which was not very effectual” and “it’s just that we’ve moved from an unwieldy committee to a single officer” [92]. The content of documents obtained through a Freedom of Information request (and have been made available to the public online) with respect to the Department of Immigration and Border Protection’s decision-making rationale around the appointment of a new Independent Health Advisor role, to replace the Immigration Health Advisory Group, are more telling [90]. In these documents, a major “key issue” is had with some Immigration Health Advisory Group’s members’ ability “to provide health advice independent of other interests”:

[3]… The performance of public duty in an independent role such as IHAG [Immigration Health Advisory Group] can have the potential for conflicts of interest to arise for some members. In some cases, these private and professional interests may be difficult to reconcile with the public duty arising from the discharge of an independent advisory role.

[4]. These conflicts are arising for some members from the natural professional interests and obligations that some members have (including, in some cases, public and media comment related to issues under consideration by IHAG [Immigration Health Advisory Group]), as well as from the interests of the professional organisation that nominated the member to IHAG in the first place. These actual and potential conflicts also present challenges to sharing information on policy and operational activities that are becoming increasingly problematic [90].

The effective silencing of the Detention Health Advisory Group and then the Immigration Health Advisory Group by the Australian Government is concerning, but not unusual. Health care providers in detention centres, for example, subject to the terms of their employment are forbidden to serve in advocacy roles for their clients. Professor Ziegler recently highlighted that health professionals in offshore detention facilities will be breaking the law if they report knowledge or strong suspicion of emotional, physical or sexual mistreatment of child detainees; yet, they are also professionally and ethically obliged to do so [69]. Indeed, if they did not report such knowledge in other settings in Australia, they would be in breach of the law. As Professor Ziegler informs:

It isn’t surprising to learn that colleagues have declared a willingness to risk gaol rather than fail to uphold professional and moral obligation to report mistreatment. This draconian legislation is hard to get your mind around. It’s reminiscent of the behaviour of totalitarian regimes [69].

In addition, healthcare providers working in immigration detention facilities are implicitly held to a lesser standard than their medical colleagues: working within centres subject to Commonwealth immigration law, they are outside the realms, regulations and oversight of state and territory health law and policy. This makes such healthcare providers unaccountable to the principles, social norms and institutions, which typically oversee and regulate their practice [18]. However, the Australian Government’s systematic suppression of the voices of detention healthcare staff is most evident in the UN Special Rapporteur on the Human Rights of Migrants’ recent refusal to visit Australia’s offshore detention facilities for the reason that if staff spoke to him they could not be guaranteed legal immunity by the Australian Government [94]. Such measures have unsurprisingly resulted in the muting of any full and frank reporting on detainees’ health status, the type and level of the available social determinants of health, and the availability, accessibility, acceptability and quality of health care services.

## Recommendations

The prevention, protection and promotion of the health and well-being of individuals and populations, which are the core business of public health [95], requires parallel and complementary efforts to maintain and protect social ethics and the human right to health. In order to achieve this, we need to understand the health needs and problems of the target group and design evidence-based health and intersectoral policies that reflect the Australian Government’s obligations under international law. As this paper has confirmed, there exists a strong evidence base as to the health and human rights breaches experienced by detainees, especially in Australia’s offshore immigration detention facilities. We also know that successive Australian Governments, in restricting research and information related to asylum seekers, have purposively ensured that statistical and other forms of data on this populace is inaccurate and this practice has been an effective strategy for rendering invisible the health needs and inequalities in this already vulnerable group [96].

While we recognise the importance of ongoing research into the health and well-being of individuals in immigration detention, unlike other refugee health commentators [18, 97, 98], however, we do not stress the impetus for further research into the plight of detainees at the hands of the Australian Government and its agents. Instead, we believe it is now time to translate the large evidence base into policy and action [99]. The need for the translation of the research on refugee health into cogent policy is crucial. It is because it is highly unlikely, in light of the contractual silence and lack of transparency that overhangs the operation of Australia’s offshore detention facilities, that the Australian Government would actively support an open and participatory health research process to take place in its detention centres at present.

Under international law, Australia is bound in good faith to uphold its obligations under the treaties it has ratified. By choosing to ignore its legal obligations and failing to report on the well-being of people seeking asylum legally under Australia’s protection, the government is aligning Australia with other draconian states that have scant regard for transparency in human rights or international law. To force the government to comply with its legal obligations and reverse current regressive practices, a comprehensive shift in the attitudes of the Australian public toward asylum seekers is needed. As health professionals, we must protest when as now, government policy deliberately breaches the right to health, fails to uphold its international legal commitments and ignores a large body of cogent evidence which points to the causal links between detention and poor health outcomes [16, 100, 101]. The continuation of such a status quo is clearly unacceptable. While a public health perspective is only one aspect of a complex social and political issue, public health professionals have a role to play in educating the public and facilitating shifts in their attitudes and ensuring that the Australian public understands this is a legal issue: at the end, it is the power of the vote that determines whether or not the right to health is realised for people seeking asylum. Here we propose four recommendations, grounded in the need for a greater level of accountability and collaboration among health professionals.

1. Build coalitions and engage the public
  Health professionals in the past successfully advocated for the establishment of the now disbanded Detention Health Advisory Group and Immigration Health Advisory Group, and we must unite again to ensure the right to health for people seeking asylum. The recent calls by 14 peak health bodies, many of whom were represented in the Detention Health Advisory Group and Immigration Health Advisory Group, are a start on this path to action [102]. We must advocate for the end of detention, especially of children and a shift to community-based processing, not only based on human rights and Australia’s legal obligations but also on the grounds of cost-effectiveness, as mandatory detention is typically the most expensive form of processing for taxpayers. A recent Commission of Audit revealed that estimates of the yearly cost of holding one asylum seeker, or “illegal maritime arrival” in the language of the Government, in onshore detention increased from AUD $179,000 in 2011–2012 to AUD $239,000 in 2013–2014 [103]. The costs of offshore processing are even higher given the cost of delivering services to remote locations and the ongoing health costs of mandatory detention are huge. The National Commission of Audit, for example indicated that the cost of holding one person in offshore detention for 12 months in 2013 to be over $400,000 [103]. In 2014–2015, the combined budget expenditure for the detention centres in Nauru and Papua New Guinea (excluding aid contributions) were approximately AUD $1.2 billion dollars, which was broken down as AUD $630 million for Papua New Guinea and AUD $582 million for Nauru [104]. While coalitions with partners in health are important, non-traditional or non-health alliances are essential to presenting common messages and changing public opinion in order to secure policy change. This must include working with a range of media to change the current discourse and social construction of people who seek asylum. People who seek asylum are not acting against the law and they are not “illegal immigrants” or “queue jumpers”. Rather, they are people fleeing insecurity and persecution, often religious or other persecution, often at the hands of their own governments, and who are exercising their right to protection—a right that Australia has pledged to honour. Research suggests that refugees contribute significantly to host societies bringing needed skills, services and entrepreneurship and demand for host country products, and we need to promote these positive stories and address the fears of those who have discriminatory attitudes toward asylum seekers. A sustained and coordinated effort to redefining the problem is critical in changing community opinion and providing a policy window for change.
2. Secure and ensure greater resources for United Nations High Commissioner for Refugees
  Despite the rhetoric, there is no “queue” that asylum seekers trying to reach Australia by boat are jumping. The key to an asylum system and in preventing its misuse is an efficient, transparent and fair processing system. Australia and other wealthy nations must provide more resources and political commitment to the UN High Commissioner for Refugees to allow more transparent and fast processing of asylum seeker applications particularly in Indonesia and Malaysia, and especially for asylum seekers in protracted situations. For this to be effective, however, it also means that wealthier countries must agree to, and offer, resettlement to people identified by the UN High Commissioner for Refugees as refugees. Supporting and ensuring this international asylum seeker system works efficiently could prevent people embarking on perilous journeys and the need for detention in third countries.
3. Immigration law reform and Human Rights Act in Australia
  As recently seen in the 2016 Federal election, there is limited political will to consider or engage in dialogue with the Australian population by the Australian Labour or Liberal parties and their supporters, with regard to alternative options to offshore processing or amnesty for those currently in offshore detention centres. We must join with like-minded groups and advocate for immigration law reform in Australia and to incorporate international treaties such as the International Covenant on Economic, Social and Cultural Rights that includes the right to health in Article 12, into domestic law. In addition to carrying out legal reform from within, Australia also needs to take the lead in developing a regional protection framework to improve human rights in the region, especially with countries that are not signatories to the UN Refugee Convention and have not offered safety services or facilities for asylum seekers. This should include developing asylum laws and procedures for refugee status determination within an international human rights framework [105], and to provide safe routes and protection for people seeking asylum. Indeed, as Human Rights Watch has highlighted, Australia can use its long-standing development aid and economic ties within the region to advocate for an improvement in human rights standards as part of its bilateral and multilateral relations, and through example [106].
4. Accountability for the health of asylum seekers through inclusion in Sustainable Development Goal Statistics
  With the post-2015 Sustainable Development Goals now agreed, there is a debate about what these newly released development goals mean both for Australia and the region. As elsewhere, this includes debate on the minimum data set to measure progress against the Sustainable Development Goals. While the Sustainable Development Goals have not included a target to reduce the number of refugees and asylum seekers, the focus on reducing inequities (Goal10) and the post-2015 development agenda to “leave no one behind” must include a focus on people seeking asylum. Indeed, target 7 for the Sustainable Development Goal 10, aims to “Facilitate orderly, safe, regular and responsible migration and mobility of people, including through the implementation of planned and well-managed migration policies” [107]. While there is no agreement as yet on how to measure this and definitions are contested [108], monitoring of the Sustainable Development Goals must include evaluation of migration policies and their implementation by the UN Member States with respect the health and well-being of people seeking asylum and others fleeing from inhumane and degrading treatment [109] that reside within such UN Member States borders, or in the case of Australia, with respect to those individuals in offshore detention facilities overseen by the Australian Government.

## Conclusions

Health and rights are “political categories” that are often problematic for both health advocates and health policy makers. Often unhelpful and misplaced dichotomies are developed in response, especially by politicians: refugee health versus Australian border security; inappropriate funnelling of resources into refugee detainee’s health versus the health and well-being of marginalised Australians in under-resourced settings. Yet, the right to health is *not* a right to health for some, it is a right to health *for all*, and particularly the most vulnerable and marginalised among us. We submit it is the responsibility of public health professionals to support the right to health *for all*, to both provide and bring the evidence base of right to health (and other inter-related rights) violations to the Australian public’s attention and to advocate for policies and programmes that seek to overcome these breaches of basic human rights.

Unless the Australian Government’s practices that jeopardise the right to health of people seeking asylum are challenged, a disturbing complicity on violation of human rights will continue to be attached to Australia, including its peoples. There are likely to be long-term consequences, not only for the affected individuals but also for Australian society more generally if we continue to accept the Government’s circumvention of international law and health and human rights.

Both those actively engaged in public health and those in human rights recognise that discrimination and other violations of human rights directly impact on health and well-being, and that they must deal directly with the underlying societal values that largely determine who lives and who dies, when and of what [110].

## Abbreviations

UN, United Nations
